# Aerophagy and Flatulent Dyspepsia

**Published:** 1909-04-17

**Authors:** 


### Medicine.

### AEROPHAGY AND FLATULENT DYSPEPSIA.

Aerophagy, or the swallowing of air, is undoubtedly at the root of certain cases of flatulent dyspepsia, and if the habit of air-swallowing can be broken the dyspeptic symptoms are relieved at once in a way that cannot be brought about by the use of drugs and dietaries.

There are two clinical groups of air-swallowers, the involuntary and the voluntary. In the involuntary form the condition manifests itself by veritable crises of eructations of wind lasting more or less long, recurring more or less frequently, yet capable neither of being evoked at will, nor stopped, nor moderated. It is commoner in patients of nervous temperament, a convulsive manifestation analogous to hysterical hiccough or to nervous cough, without any direct relation to gastric disorders. It is not of this type that we would speak here. In the second form the air-swallowing is under the control of the will. It is to be observed in neurotic gastropaths who have developed the habit, performing the act repeatedly and intentionally in the idea of relieving certain gastric sensations in this way. A great deal of flatulent dyspepsia and of the abundant belching of wind, sometimes almost at will, are due to this cause.

The following account is based on a case recorded very fully by Soupault. The patient was a man aged 42, who sought medical advice about flatulent dyspeptic symptoms that had been troubling him for over three years. He had never had any major illness except syphilis at 20, which had been cured without trouble. His general condition was satisfactory except for a drawn aspect about his face. He stated that he had decreased in weight to the extent of over a stone in three years. None of the stigmata of neurosis nor of neurasthenia were to be noted though the man was a little tired.

When questioned in detail, it turned out that although the gastric symptoms had been more noticeable during the last three years, they had really started ten years ago. No adequate cause for their commencement could be stated, though the patient admitted that he had more than once taken alcohol to excess, and that he was in the habit of taking potassium iodide from time to time. The first gastric symptoms were a sense of weight rather than of actual pain during digestion, and slight regurgitations and repeatings; even these slight symptoms were not always present. They were produced, as a rule, whenever he exceeded in his dietary, or when he was physically or mentally fatigued. During the last three years there had been an almost sudden increase in the symptoms. After a brisk purge one day violent epigastric pains and discomfort set in, and from that moment the flatulent syndrome appeared and persisted.

On admission he was a constant sufferer from a feeling of weight and discomfort in the epigastrium, notably augmented immediately after a meal, but without any actual acute pain, colic, or burning sensation. After eating, the stomach became blown up. A little later the heart was apt to palpitate, and the patient suffered from hot flushes over his body and a feeling of being intellectually torpid. Appetite was none the less excellent; the patient used to eat plenty, and he looked well enough nourished. The other, and, indeed, the main, symptom was the extraordinary flatulence. Belching of wind occurred all day long: on waking in the morning, during the day, both before and after meals, on going to bed, and even when he was awakened from any cause during the night. During actual sleep there were no belchings.

The eructations of wind were always produced in the same way. First of all the patient closed his mouth hermetically, then, making a series of combined movements with the muscles of the mouth, the palate, and the pharynx, he drew in through his nostrils a quantity of air, which he forthwith swallowed and compelled to go into his stomach with a peculiar sound. Six or eight of these movements would be made in rapid succession. Inspection of the epigastric region enabled one to see the stomach swelling progressively until it became so distended that its outlines could be clearly seen. After a certain number of the air-swallowing acts the patient opened his mouth, and the stomach contractions forced out some of the air, giving rise to a loud belch. For the moment there was relief to the abdominal symptoms, but shortly afterwards the air-swallowing would be repeated, and then there would be further belchings, and so on.

It was easy to make certain that the air was forced into the œsophagus by a veritable act of deglutition; in other words that the case was one of aerophagy. Auscultation of the œsophagus through the spine behind, and of the stomach through the epigastrium, allowed of the downward movement of the air being easily followed. Moreover—and this constitutes the essential point in the treatment of these cases—by placing a cork or other suitable body between the teeth so as to prevent deglutition, both flatulence and belching ceased forthwith.

It is needless to go into this case in greater detail here. So extreme a degree of flatulence from aerophagy is doubtless rare, but minor degrees, if watched for, are found to be comparatively common. Aubert has pointed this out, remarking at the same time that numbers of persons who are not really flatulent, and even not dyspeptic, intuitively swallow air to assist their eructations. It is the exaggeration of this phenomenon that gives rise to the clinical picture of flatulent dyspepsia. The amount of gas brought up varies from a pint at a time down to quite small volumes. The explanation of this is simple enough. Different patients suffering from flatulent dyspepsia swallow air with very different degrees of avidity. Some make as many as five, six, or more repeated movements of deglutition, distending their stomachs to a marked degree before allowing the gas to escape. Others make only two or three swallowings between each eructation. Others again only one. The intensity, the violence of the movements are not always identical; hence the amount of gas brought back also varies considerably. It even happens sometimes that the air ingested is so small in amount that it does not go down so far as the stomach, and there arises a condition of simple œsophageal eructation. Even though the aerophagy is primarily voluntary, it often takes place quite unconsciously after a while, the constant repetition of the act having created a sort of habit.

When patients are closely questioned, it appears that they have suffered from some degree of dyspepsia before flatulence develops. The eructations of gas are seldom, if ever, a phenomenon of the onset of dyspepsia. Conversely, many patients swallow air and can eructate it at will without suffering from any dyspeptic symptoms at all. As a rule the aerophagy of dyspeptic cases is worst after food. A meal produces uncomfortable epigastric sensations, and these in turn provoke the swallowing of air. Flatulence results. According to the type of the primary dyspepsia the flatulence reaches its maximum either shortly after the meal or a long time after it, or even after only a few mouthfuls of food have been eaten. Everything that tends to aggravate the dyspepsia also, broadly speaking, increases the aerophagy, and consequently the flatulence. Physical fatigue may do so in some cases, emotional disturbances in others, or indiscretions of any kind.

If this be the pathology of the flatulence in a given case, it is clear that the giving of antiseptic remedies is not likely to relieve it. Antiseptic drugs are prescribed with the idea of inhibiting the production of gas by fermentative micro-organisms. If the gas is derived from the air by a process of swallowing, it is clear that some other line of treatment is required. There are, of course, many cases of flatulence from fermentative changes in the gastric contents besides those due to aerophagy; but when the trouble is due to the latter, neither carminatives, acids, nor alkalies are likely to do half so much good as is the prevention of aerophagy. Many patients can cure themselves of the habit very quickly when its nature is pointed out to them; it may be a great assistance to them if they carry with them a cork or other similar body that they can place between their teeth whenever they catch themselves in the act of swallowing air, so as to prevent the continuance of the aerophagy.

